# The Accuracy of Spot Sign in Predicting Hematoma Expansion after Intracerebral Hemorrhage: A Systematic Review and Meta-Analysis

**DOI:** 10.1371/journal.pone.0115777

**Published:** 2014-12-26

**Authors:** Fei-Zhou Du, Rui Jiang, Ming Gu, Ci He, Jing Guan

**Affiliations:** Department of Radiology, Chengdu Military General Hospital, Chengdu, China; University of Michigan, United States of America

## Abstract

**Purpose:**

The role of spot sign on computed tomography angiography (CTA) for predicting hematoma expansion (HE) after primary intracerebral hemorrhage (ICH) has been the focus of many studies. Our study sought to evaluate the predictive accuracy of spot signs for HE in a meta-analytic approach.

**Materials and Methods:**

The database of Pubmed, Embase, and the Cochrane Library were searched for eligible studies. Researches were included if they reported data on HE in primary ICH patients, assessed by spot sign on first-pass CTA. Studies with additional data of second-pass CTA, post-contrast CT (PCCT) and CT perfusion (CTP) were also included.

**Results:**

18 studies were pooled into the meta-analysis, including 14 studies of first-pass CTA, and 7 studies of combined CT modalities. In evaluating the accuracy of spot sign for predicting HE, studies of first-pass CTA showed that the sensitivity was 53% (95% CI, 49%–57%) with a specificity of 88% (95% CI, 86%–89%). The pooled positive likelihood ratio (PLR) was 4.70 (95% CI, 3.28–6.74) and the negative likelihood ratio (NLR) was 0.44 (95% CI, 0.34–0.58). For studies of combined CT modalities, the sensitivity was 73% (95% CI, 67%–79%) with a specificity of 88% (95% CI, 86%–90%). The aggregated PLR was 6.76 (95% CI, 3.70–12.34) and the overall NLR was 0.17 (95% CI 0.06–0.48).

**Conclusions:**

Spot signs appeared to be a reliable imaging biomarker for HE. The additional detection of delayed spot sign was helpful in improving the predictive accuracy of early spot signs. Awareness of our results may impact the primary ICH care by providing supportive evidence for the use of combined CT modalities in detecting spot signs.

## Introduction

Intracerebral hemorrhage (ICH) is the cause of up to 15% of all strokes and carries a poor prognosis. It represents a considerable unmet medical need despite recent advances [1], [2]. Hematoma expansion (HE) has been recognized as an independent predictor for clinical deterioration, mortality, and poor outcome after ICH [3]–[5]. However, hemostatic drugs were not verified to restrict HE in previous trials [2], and they were criticized to include a great proportion of patients who may not have benefited from hemostatic treatment because their bleeding had already ceased [6].

It is not easy to determine which population is likely to develop HE. Recently, spot sign detected by CT angiography (CTA) has emerged as a potential predictor for HE after primary ICH (primary ICH). However, the underlying pathophysiology mechanisms remained unclear with a series of possible explanations, including Charcot-Bouchard microaneurysms, pseudoaneurysms, fibrin globes, and breakdown of blood-brain barrier [7]–[9].

Original studies on spot sign differed in population, imaging techniques, radiographic criteria, and definition of outcomes, with a wide range of predictive values. Two recent reviews have summarized the role of CTA spot sign in primary ICH [10], [11]. However, no published report has explored the predictive accuracy of spot sign by using a meta-analytic approach. Thus, we carried out this study aiming to evaluate the accuracy of spot sign in predicting HE after primary ICH.

## Methods

### Search Strategy

The supporting PRISMA checklist is available as supporting information; see S1 PRISMA Checklist. We conducted this meta-analysis according to the PRISMA statement [12]. We systematically searched Pubmed, Embase, and the Cochrane Library up to August 2014, restricting to English language. Search terms and keywords were grouped in the following search strategy: (“spot sign” OR “contrast extravasation” OR “postcontrast leakage” OR “computed tomography angiography” OR “CTA” OR “postcontrast CT”) AND (“intracerebral hemorrhage” OR “intracerebral hematoma” OR “intracranial hematoma” AND (“hematoma expansion” OR “hematoma growth” OR “hematoma enlargement” OR “recurrent bleeding”). Further, we searched the reference lists of relevant articles for additional studies.

### Definition

Definitions of spot sign varied across studies [10]. Currently, spot sign was well-acknowledged as the foci of enhancement within the intracranial hematoma, detected on CTA source images [10]. We accepted a broad concept of spot sign that was detected by various CT modalities, including CTA, CTP and PCCT. Accordingly, we categorized spot signs into early spot signs and delayed spot signs. The early type was detected by the first-pass CTA. The delayed type was detected by the second-pass CTA, PCCT, or CTP. The first-pass CTA images were normally acquired in the arterial phase within 30 seconds after contrast injection. The second-pass CTA, namely the delayed CTA, was normally performed between 40 seconds to 3 minutes after contrast injection, which assessed the spot sign during the venous phase [10]. We accepted the definition of HE as an increase in ICH volume of >6 ml or >30% from the baseline ICH volume [8], [14].

### Study Selection

Two reviewers (FZD and MG) screened titles and abstracts to identify eligible studies. Studies were included when meeting the following criteria: (1) original research; (2) investigated spot sign on CTA in patients with primary ICH; (3) reported data of HE in spot-sign negative and spot-sign positive groups; (4) reported clear definition of HE, which at least showed an increase in ICH volume of >6 ml or >30% from the baseline ICH volume. We excluded studies of secondary ICH resulting from trauma, tumor, intracranial aneurysm, arteriovenous malformation, or other causes. Studies examining spot signs by MRI were excluded.

### Data Extraction and Quality Assessment

Two assessors (FZD and RJ) reviewed the full text of selected studies. Data were extracted independently in standardized forms. When duplicate cohorts were detected, the most informative cohort was included. The following items were extracted: author, year, study design, sample size, gender, CT modalities, CT type, definition of HE, time from onset to CTA, time from initial CT to HE assessment, and blinded assessment. Raw data were extracted into 2×2 contingency tables of positive and negative spot sign against clinical outcomes. Given the diagnostic feature of our research, selected papers were critically appraised through the QUADAS tool [13]. The reference standards in our study were clinical outcomes. So, we omitted one item on the time period from index test to reference standard, as the reference standard diagnoses are largely reached within a short period, thus eliminating the possible delayed verification bias [14].

### Statistical Analysis

The Meta-Disc software 1.4 (Clinical Biostatistics, Ramony Cajal Hospital, Madrid, Spain) was used to perform analyses of predictive accuracy [15]. The sensitivity, specificity, positive likelihood ratio (PLR), negative likelihood ratio (NLR) and diagnostic OR (DOR) were calculated [16]. Although PLR above 10 or NLR below 0.1 represented the most conclusive predictive value, we accepted PLR above 5 or NLR below 0.2 as satisfactory predictive values [17], [18]. The DerSimonian and Laird's random-effects model was employed for pooling the results. The heterogeneity between studies was assessed qualitatively by Cochran's Q test, and quantitatively by I^2^ statistic. A P value of less than 0.05 by Cochran's test indicated significant heterogeneity. A study with an I^2^ greater than 50% suggested substantial heterogeneity. The threshold effect was indicated when a “shoulder arm” pattern was present, or when the Spearman correlation coefficient in the threshold analysis showing a strong positive correlation [15]. Summary receiver operating characteristic (SROC) curves were further constructed by using the Moses-Shapiro-Littenberg method [19]. The Q*index and area under the curve (AUC) were calculated [20]. Because likelihood ratios, DORs, and SROC curves had the advantage of considering both the sensitivity and specificity data, they are more valuable for evaluating the diagnostic accuracy than sensitivity or specificity.

The publication bias was visually inspected by Funnel plot and statistically calculated by Deek's test [21]. The STATA software (version 12.0; Stata Corporation, College Station, Texas) was employed to explore the publication bias. We inferred several potential sources of heterogeneity *a priori*: (1) study design (prospective or retrospective); (2) sample size (<100 or ≥100); (3) results interpretation (blinded assessment or non-blinded assessment of radiographic features); (4) time to CTA (<6 h or ≥6 h). Subgroup analyses and univariate meta-regression were conducted to explore heterogeneity. A threshold of P <0.1 was defined for publication bias or heterogeneity existed.

## Results

### Literature Search and Study Characteristics

**Figure 1:**
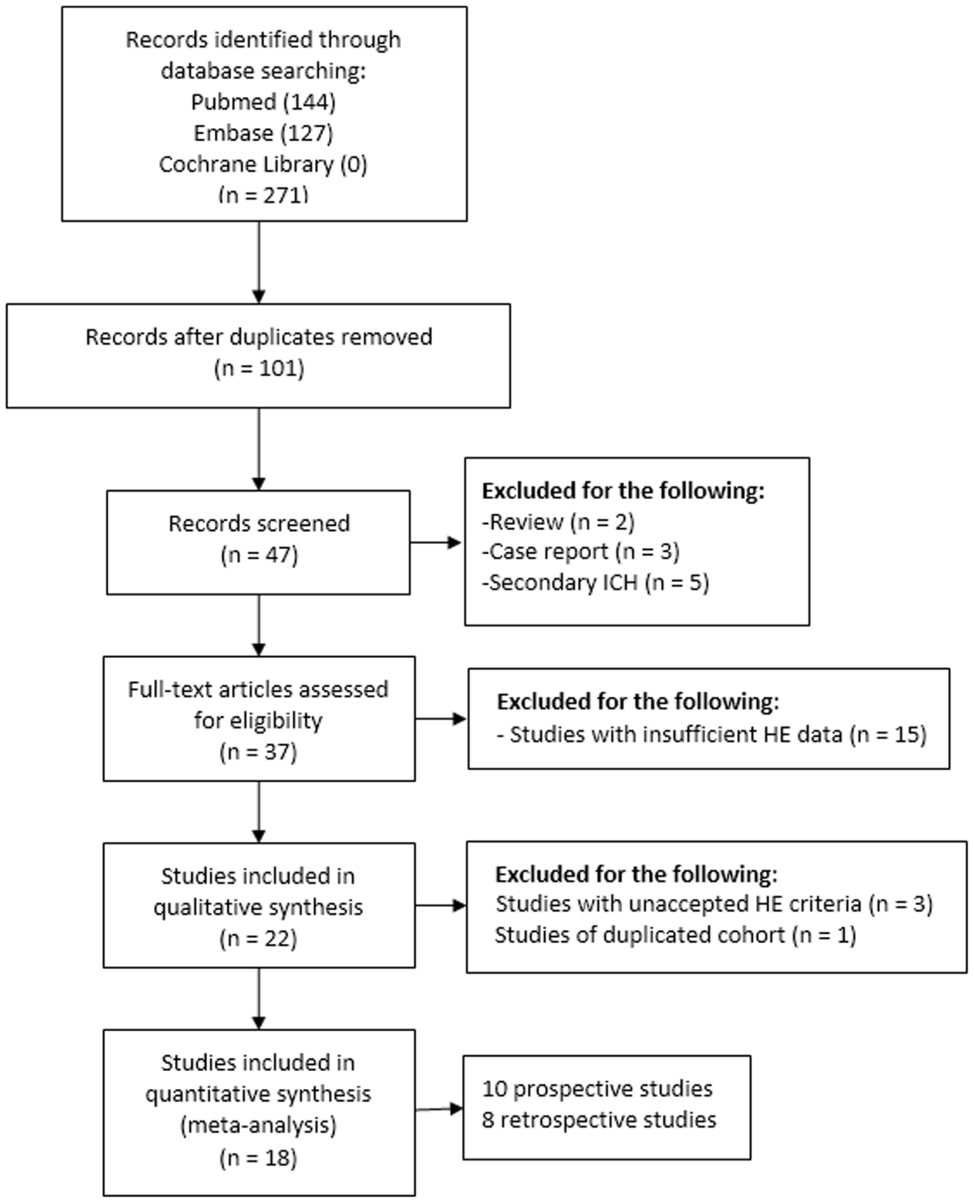
The flowdiagram for selection of eligible studies.

We initially retrieved 271 articles. Then we identified 47 relevant original studies after removal of duplications. Twenty-eight studies were further excluded, involving 5 studies of secondary ICH [22]–[26], 3 case reports [27]–[29], 2 review article [10], [11], 15 studies with insufficiently detailed records of HE data [7], [30]–[44], and 3 studies with definition of HE contradicting with our HE criteria [45]–[47]. Then we carefully assessed studies carried out by the same institutions [9], [48]–[53], and data from two identical cohorts were incorporated together [48], [49]. Thus, 18 studies were included into the meta-analysis, including 10 prospective studies, and 8 retrospective studies (Fig. 1). The study characteristics were summarized in Table 1. Rodriguez-Luna et al. performed post hoc analysis of the previous PREDICT study, with both early spot signs and delayed spot signs dynamically investigated . As the early spot signs were already included from the original PREDICT study [52], we only included the data of delayed spot signs in the post-hoc report [53]. The characteristics of studies were shown in Table 1. According to the modified 13 items QUADAS tool, most studies were of high quality (S1 Table). Notably, the criterion satisfied least was blinded assessment of spot sign and hematoma expansion.

**Table 1:** Characteristics of included studies.

Author, year	Study design	Location	Sample size	Male, %	Imaging modality	CT type	Definition of HE	Time from onset to CTA (h)	Time from initial CT to HE assessment (h)	Blinded assessment
Wada (2007)	Prospective	Canada	39	67	First-pass CTA; PCCT	4-slice; 64-slice	>6 ml or>30%	<3	<48	NA
Goldstein (2007)	Retrospective	USA	104	53	First-pass CTA	Helical	>33%	<48	<48	Yes
Delgado (2009)	Retrospective	USA	367	58	First-pass CTA; second-pass CTA	16-slice helical; 64-slice helical	>6 ml or>30%	Mean: 7.4	<48	Yes
Ederies (2009)	Retrospective	Canada	61	67	First-pass CTA; PCCT	4-slice; 64-slice	>6 ml or >30%	<6	<24	Yes
Delgado (2010)	Retrospective	USA	573	54	First-pass CTA; second-pass CTA	16-slice helical; 64-slice helical	>6 ml or >30%	Mean: 7.4	<48	Yes
Evans (2010)	Retrospective	Canada	59	59	First-pass CTA; PCCT	4-slice; 64-slice	>6 ml or >30%	Mean: 4.5	<24	NA
Park (2010)	Prospective	Korea	110	63	First-pass CTA	64-slice helical	>6 ml or >30%	<24	<48	NA
Wang (2011)	Retrospective	China	312	61	First-pass CTA	64-slice helical	>6 ml or >30%	<3	<24	NA
Li (2011)	Prospective	China	139	68	First-pass CTA	16-slice	>12.5 ml or >30%	<6	<24	Yes
Demchuk (2012)	Prospective	Multicenter*	228	57	First-pass CTA	No specifications	>6 ml or >33%	<6	<24	Yes
Brouwers (2012)	Retrospective	USA	391	55	First-pass CTA	NA	>6 ml or >33%	Median: 6	<48	Yes
Junior (2013)	Prospective	Brazil	65	62	Dynamic CTA	64-slice	>6 ml or >33%	Mean: 10.9	NA	NA
Rizos (2013)	Prospective	Germany	101	61	First-pass CTA	16-slice	>6 ml or >33%	<6	Median: 19	Yes
Romero (2013)	Prospective	USA	131	61	First-pass CTA	64-slice helical	>6 ml or >33%	<24	<24	NA
Sun (2013)	Prospective	China	112	64	First-pass CTA; CTP	16-slice multidetector	>6 ml or >30%	<6	<24	Yes
Brouwers (2014)	Prospective	USA	817	56	First-pass CTA	NA	>6 ml or >33%	Median: 5.0	Median: 18	Yes
Hotta (2014)	Retrospective	Japan	323	58	First-pass CTA	40-slice	>12.5 ml or >33%	<24	<24	NA
Rodriguez-Luna (2014)	Prospective	Multicenter*	371	NA	Dynamic CTA	NA	>6 ml or >33%	<6	<24	Yes

CTP, CT perfusion; HE, hematoma expansion; NA, not available; PCCT, post-contrast CT; SS, spot sign.

*Multicenter included Canada, Spain, Germany, Poland, India, and USA.

### First-pass CTA

**Figure 2:**
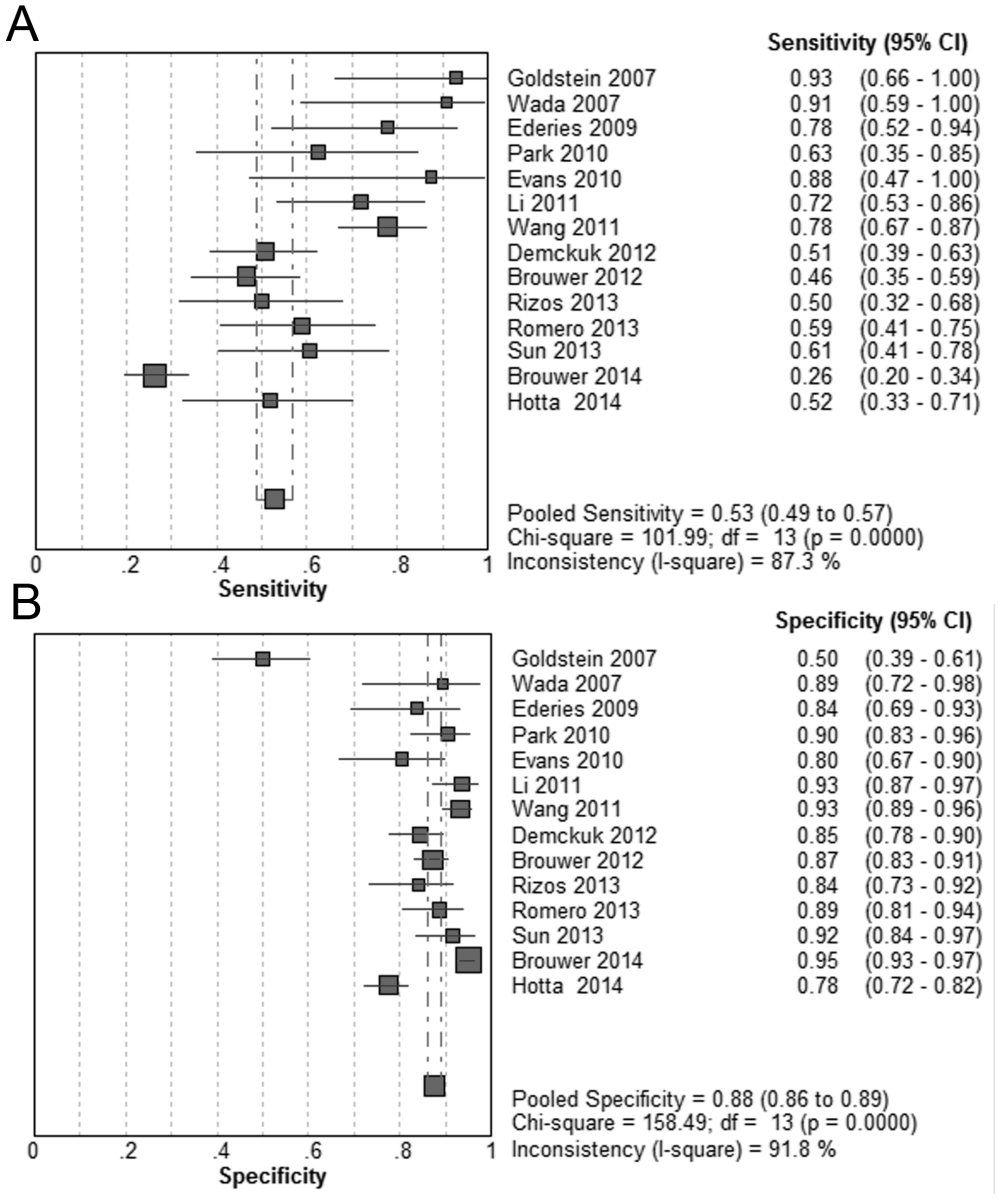
Summary of sensitivity and specificity of first-pass CTA spot signs in predicting hematoma expansion.

**Figure 3:**
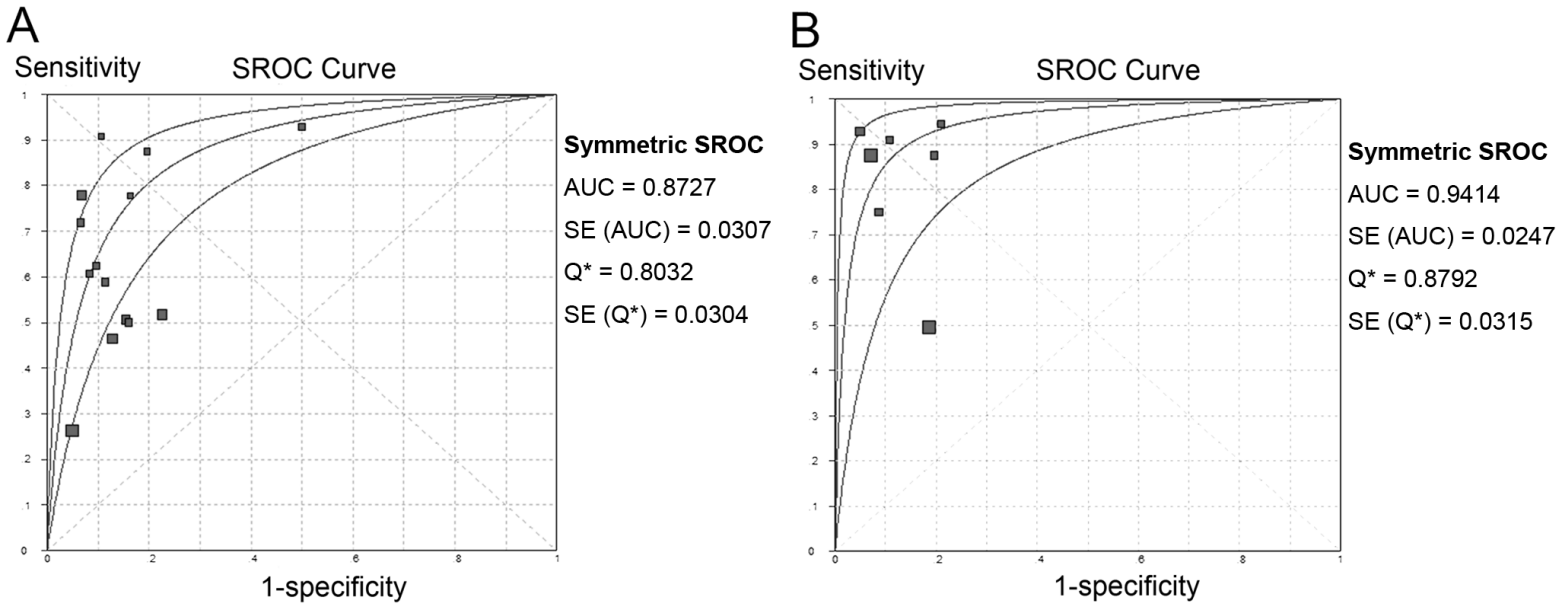
Summary of SROCs of spot signs on first-pass CTA or combined CT modalities for predicting hematoma expansion. The curve is the regression line that summarizes the overall predictive accuracy. The upper and lower curves represent confidence intervals. Squares indicate individual study estimates of sensitivity and 1-specificity. The size of each square is proportional to the sample size of the corresponding study. Q*, the maximum joint sensitivity and specificity on a symmetric ROC curve; SE (AUC), standard error of AUC; SE (Q*), standard error of the Q* value. (A) first-pass CTA; (B) combined CT modalities.

Fourteen relevant studies were identified. Spot sign was significantly associated with increased risk of HE (DOR  = 11.84; 95% CI, 7.35–19.05; *P*<0.05; I^2^ = 71.6). The pooled sensitivity was 53% (95% CI, 49%–57%) with a specificity of 88% (95% CI, 86%–89%) (Fig. 2). The summary PLR was 4.70 (95% CI, 3.28–6.74) and the overall NLR was 0.44 (95% CI, 0.34–0.58). Significant heterogeneity was revealed for all results (*P*<0.05). The SROC curve yielded an AUC of 0.87 (Fig. 3A). (Table 2).

**Table 2:** Pooled results of spot signs detected by different CT modalities for predicting hematoma expansion.

Imaging modality	Study, n	SEN (95% CI)	SPE (95% CI)	PLR (95% CI)	NLR (95% CI)	DOR (95% CI)	AUC (SE)	Q (SE)
First-pass CTA	14	53% (49%–57%)	88% (86%–89%)	4.70 (3.28–6.74)	0.44 (0.34–0.58)	11.84 (7.35–19.05)	0.87 (0.03)	0.80 (0.03)
First-pass CTA & Delayed CTA	4	65% (57%–73%)	88% (85%–91%)	6.43 (2.02–20.50)	0.29 (0.08–1.02)	22.40 (2.47–203.26)	0.99 (0)	0.97 (0.01)
Post-contrast CT	2	41% (24%–61%)	93% (84%–98%)	5.38 (2.05–14.13)	0.64 (0.47–0.87)	8.74 (2.64–28.98)	0.50 (0)	0.50 (0)
First-pass CTA & Post-contrast CT	3	92% (78%–98%)	82% (74%–88%)	4.89 (3.29–7.27)	0.10 (0.04–0.31)	52.62 (14.46–191.51)	0.94 (0.05)	0.88 (0.06)
Combined CT modalities	7	73% (67%–79%)	88% (86%–90%)	6.76 (3.70–12.34)	0.17 (0.06–0.48)	43.51 (10.03–188.81)	0.94 (0.02)	0.88 (0.03)

AUC, area under curve; DOR, diagnostic odds ratio; NLR, negative likelihood ratio; PLR, positive likelihood ratio; SE, standard error; SEN, sensitivity; SPN, specificity.

The publication bias was represented by examining studies of first-pass CTA. No publication bias was revealed by visual inspection of the funnel or by the Deek's test (*P* = 0.21). The source of heterogeneity was explored by subgroup analyses. We could not establish the subgroup of time to CTA due to heterogeneous data. Subgroup analyses were performed in terms of the stratification of study design, sample size, and blinded assessment (Table 3). Notably, studies without blinded assessment of spot signs produced DOR estimates about twofold higher than studies of blinded assessment. Studies with sample size below 100 produced DOR estimates that were about 2.6 times higher than studies with sample size over 100. The univariate meta-regression was further carried out, whereas indicating no statistical significance for study design, sample size, or blinded assessment.

**Table 3:** Subgroup analyses relating to the diagnostic odds ratio (DOR) of first-pass CTA for hematoma expansion.

Subgroups	Hematoma expansion		
	Studies, n	DOR (95% CI)	I^2^, %
Study design			
Prospective	8	10.94 (6.58–18.19)	58.0
Retrospective	6	12.79 (4.70–34.79)	82.6
Sample size			
≥100	11	10.41 (6.28–17.27)	75.2
<100	3	26.92 (9.44–76.74)	0
Blinded assessment			
Yes	8	8.91 (5.76–13.80)	51.1
None/Unknown	6	17.56 (6.25–49.36)	79.8

### Delayed CTA

Four articles were pertinent, including two articles of second-pass CTA [48], [49], and two studies of venous phase CTA [53], [54]. Data of the second-pass CTA were combined together due to duplicate cohort. The predictive accuracy of spot signs detected on delayed CTA images alone could not be calculated because of insufficient subgroup data. All studies reported the pooled results of spot signs jointly detected by the early and delayed CTA. Spot sign was significantly associated with increasing risk of HE (OR, 22.4; 95% CI, 2.47–203.26; *P*<0.05; I^2^ = 94.3). The pooled sensitivity was 65% (95% CI, 57%–73%) with a specificity of 88% (95% CI, 85%–91%). The summary PLR was 6.43 (95% CI, 2.02–20.50) and the overall NLR was 0.29 (95% CI, 0.08–1.02). The SROC curve yielded an AUC of 0.99. (Table 2)

### Post-contrast CT

Three studies additionally reported extravasation data on PCCT [7], [8], [55]. Data of extravasation detected by PCCT alone were available in 2 studies [8], [55]. The PCCT extravasation was significantly associated with increased risk of HE (OR, 8.74; 95% CI, 2.64–28.98; *P*<0.05; I^2^ = 0). The pooled sensitivity was 41% (95% CI, 24%–61%) and the pooled sensitivity was 93% (95% CI, 84%–98%). (Table 2)

We further assessed the combined use of CTA and PCCT. Spot signs detected by either method were included, and they were significantly associated with increased risk of HE (OR, 52.62; 95% CI, 14.46–191.51; *P*<0.05; I^2^ = 0). The pooled sensitivity was 92% (95% CI, 78%–98%) and the pooled specificity was 82% (95% CI, 74%–88%). The summary PLR was 4.89 (95% CI, 3.29–7.27) and the summary NLR was 0.10 (95% CI, 0.04–0.31). The SROC curve yielded an AUC of 0.94. (Table 2)

### CT Perfusion

Only one study compared CTP with CTA, which precluded meta-analysis [56]. The sensitivity, specificity, PPV, and NPV for CTP spot-sign predicting hematoma expansion were 89.3%, 94.0%, 83.3% and 96.3%, respectively.

### Overall Combined CT Modalities

**Figure 4:**
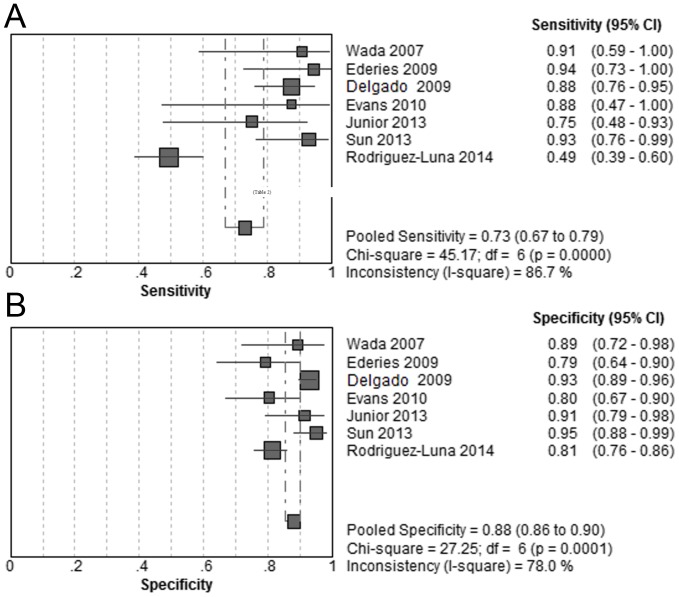
Summary of sensitivity and specificity of spot signs on combined CT modalities in predicting hematoma expansion.

To examine the strength of combined CT modalities in detecting spot sign, we pooled the results of CTA combined with any additional CT modality, namely the joint CT modalities. Accordingly, 7 studies were included. Spot sign was significantly associated with increased risk of HE (OR, 43.51; 95% CI, 10.03–188.81; *P*<0.05; I^2^ = 88.5). The pooled sensitivity was 73% (95% CI, 67%–79%) and the pooled specificity was 88% (95% CI, 86%–90%) (Fig. 4). The summary PLR was 6.76 (95% CI, 3.70–12.34) and the summary NLR was 0.17 (95% CI, 0.06–0.48). The SROC curve yielded an AUC of 0.94 (Fig. 3B). (Table 2) To explore the source of heterogeneity, we further conducted sensitivity analysis by excluding the studies one by one. When excluding study by Rodriguez-Luna et al., the pooled diagnostic OR was 76.81 (95% CI, 41.21–143.15), without evidence of heterogeneity (I^2^ = 0, *P* 0.54).

## Discussion

We are aware of one recent systematic review regarding spot sign [11]. Notably, the included criteria was quite strict, as only studies reporting clinical outcomes (prognostic scale scores or death) of patients over 18 years were included. Thus, only 6 studies were reviewed. The author failed to conduct meta-analysis to pool the predictive accuracy of spot sign. Besides, the authors only focused on first-pass CTA spot signs. In comparison, firstly, our research included more updated and comprehensive studies, with those reporting HE data rather than clinical outcome data included. We did not constrict the age criteria since most related studies reported a wide range of ages. Secondly, a meta-analysis was performed to pool the predictive accuracy of spot sign, not only with SROC curves depicted, but also with different combination of imaging modalities compared. Thirdly, we systematically explored potential biases and sources of heterogeneity, which were scant in the previous review.

Spot sign is a dynamic radiological parameter with different sensitivity and specificity, depending on the imaging delay after contrast administration [48], [55], [57]. In general, the delayed time ranges from 5 s to 40 s for first-pass CTA [8], [9], has a median of 2 to 3 minutes for second-pass CTA [48], [49], and has a range of 3 to 5 minutes for PCCT [31]. Delayed imaging (>2 min) may help detect spot sign with increased time interval during which contrast is circulating and permeating into the hematoma. The acquisition of first-pass CTA may be too quick relative to bolus injection and not permit sufficient time for spot opacification, and thus miss delayed spot sign [10].

Our pooled results revealed that the early spot sign on first-pass CTA had a moderate sensitivity and a high specificity for predicting HE. In comparison, the spot sign detected by combined CT modalities had a higher sensitivity and similar specificity for predicting HE. Especially, the combined use of first-pass CTAand PCCT showed the highest sensitivity of 92%. However, the result of this combined modality was based on only three studies and future investigations are needed to verify this finding. When calculating likelihood ratios, only the combined modalities showed a PLR above 5 as well as a NLR below 0.2, which indicated satisfactory predictive values. Our study highlighted the strength of additionally performing dynamic CTA, PCCT, or CTP, which would be beneficial for improving the predictive accuracy of first-pass CTA spot sign.

When used for patient selection, a predictive imaging biomarker needs to have a high sensitivity to minimize the risk of excluding hemorrhage patients who might benefit from timely hemostatic interventions. In fact, the low sensitivity of early spot sign has been a major concern in its application in clinical trials [10]. It has been proposed that decreased sensitivity of the spot sign may be secondary to differences in scanner speed. Most studies scanned their patients on 4-, 16-, and 64-slice scanners, and not on the new faster scanners with 128- and 320-slice scanners [58]. However, as the advanced scanner was not available in many hospitals, the combined CT modalities were worthy of consideration to improve the sensitivity.

In subgroup analysis, it seemed that studies assessed without blindness had a higher DOR than those with blinded assessment. However, results from non-blinded assessment may not be reliable. Considering possible awareness of clinical outcomes, CT readers may be more cautious when assessing patients with HE, whereas may be more unwary when assessing those without HE. In prospective trials, clinicians who were aware of imaging results may encourage interventions for arresting HE in patients with spot signs. The increased exposure suspicion bias and therapy dilution bias may weaken the genuine predictive accuracy [59], [60]. Thus, the double-blinded assessment of radiographic features as well as clinical features are pivotal to minimize bias in future trials.

It constituted a major limitation that the baseline ICH volume and time from onset to CTA varied greatly across the included studies. Subgroup analyses were not performed due to overly heterogeneous data. However, as we failed to identify significant confounding factor in meta-regression analyses, the variations in baseline ICH volume or time to CTA may contribute to the heterogeneity. Spot sign may be more easily detected in large hematoma, which also marks those patients with more severe underlying vasculopathy or coagulopathy [9]. Patients with larger baseline ICH volume might have already bled more with more poor condition [52], and small ICHs have been suggested to be associated with less hematoma expansion and better outcome [61]. Some studies showed a declined accuracy of spot sign for predicting HE as time interval prolongs, suggesting that HE occurred mainly during the first few hours following ictus [62]. However, some studies opposed, arguing that a substantial number of patients destined to suffer from HE present either late or with an unknown symptom onset time. Spot sign may accurately identify those patients irrespective of time to CTA [9], [43], [51]. In light of these controversies, interactions among these factors are worthy of further investigations. Of interest, the ultra-early hematoma growth, which represented the adjusted baseline ICH volume by onset-to-imaging time, was shown to be faster in spot-sign positive patients and better predict HE [44]. Recently, a 9-point prediction score comprised of baseline ICH volume, time to CT, CTA spot sign, and warfarin use was developed, which correlated well with HE and other outcomes [50].

Several other limitations of our study should be acknowledged. The number of included studies was still limited with small sample sizes, especially those regarding spot signs on delayed CT modalities. Few studies have implemented a joint modality to detect spot sign. For retrospective studies, the decision to perform CTA was made by the clinicians, rather than a standard protocol, and thus increased the risk of selection bias. Few studies stated that the neuro-radiologists who evaluated spot signs were blinded to the results of clinical data or non-contrast CT. The result of combined modalities was based on relatively small number of studies. Given the difficulty in sorting heterogeneous confounding factors, such as timing, scanner type, and patient population, meta-regression analyses or subgroup analyses were not performed for combined modalities. Thus, we could not preclude the possibility that the higher predictive value was in fact an artifact of these characteristics. Some other crucial clinical variables, including age, consciousness level, blood pressure level, hypertension or anticoagulation history, and coagulation parameters, were mostly unknown and not well compared between groups, and thus preclude their incorporation into subgroup analyses. Besides, it is difficult to balance the potential imaging variables, including leukoaraiosis, brain atrophy, previous stroke lesions, and complex imaging parameters [6]. One important confounding radiological factor is the kinetics of the contrast bolus, which depend on patient­related and injection­related variables, including cardiac output, concentration of the contrast medium, and injection rate. Although these factors are crucial to the spot sign appearance and magnitude, they were generally unclear and variable [1]. Finally, refinements in imaging techniques and validation of multi-itemed predictive scales, such as the spot sign score, are expected to further increase the predictive accuracy for patients who may develop HE after hemorrhage [49], [63].

Despite these limitations, our results demonstrated that spot sign appeared to be a useful imaging biomarker for predicting HE among patients with Primary ICH. Especially, the combined CT modalities showed satisfactory predictive accuracy of spot signs for hematoma enlargement. As most previous studies focused on early spot signs, we highlighted the additional value of delayed spot signs. Further studies are warranted, not only to investigate the mechanism of CE, but also to assess its power of selecting patients in clinical trials.

## Supporting Information

S1 Table **Results of quality assessment by the QUADAS tool.**
(DOCX)

S1 PRISMA Checklist (DOC)
