# Supplementary material for: The Accuracy of Spot Sign in Predicting Hematoma Expansion after Intracerebral Hemorrhage: A Systematic Review and Meta-Analysis
Source: PLoS One. 2014 Dec 26;9(12):e115777. doi: 10.1371/journal.pone.0115777 (PMC4277365; doi:10.1371/journal.pone.0115777)
Supplement: S1 Table — Results of quality assessment by the QUADAS tool. (DOCX) [file pone.0115777.s001.docx]

Supplemental Table 1. The quality assessment of included studies by modified QUADAS tool with 13-items.

|  | Representative spectrum | Clear description of selection criteria | Acceptable reference standard | Partial verification avoided | Differential verification avoided | Incorporation avoided | Index test described in detail | Reference standard described in detail | Index test results blinded | Reference standard results blinded | Relevant clinical information | Uninterpretable results reported | Withdrawals explained |
| --- | --- | --- | --- | --- | --- | --- | --- | --- | --- | --- | --- | --- | --- |
| Wada (2007) | Yes | Yes | Yes | Yes | Yes | Yes | Yes | Yes | No | Not clear | Yes | Yes | Yes |
| Goldstein (2007) | Yes | Yes | Yes | Yes | Yes | Yes | No | Yes | No | Not clear | Yes | Yes | Yes |
| Delgado (2009) | Yes | Yes | Yes | Yes | Yes | Yes | Yes | Yes | Yes | No | Yes | Yes | Yes |
| Ederies (2009) | Yes | Yes | Yes | Yes | Yes | Yes | Yes | Yes | Yes | Yes | Yes | Yes | Yes |
| Delgado (2010) | Yes | Yes | Yes | Yes | Yes | Yes | Yes | Yes | Yes | No | Yes | Yes | Yes |
| Evans (2010) | Yes | Yes | Yes | Yes | Yes | Yes | Yes | Yes | Yes | Not clear | Yes | Yes | Yes |
| Park (2010) | Yes | Yes | Yes | Yes | Yes | Yes | Yes | Yes | No | No | Yes | Yes | Yes |
| Wang (2011) | Yes | Yes | Yes | Yes | Yes | Yes | Yes | Yes | No | No | Yes | Not clear | Yes |
| Li (2011) | Yes | Yes | Yes | Yes | Yes | Yes | Yes | Yes | Yes | Not clear | Yes | Yes | Yes |
| Demchuk (2012) | Yes | Yes | Yes | Yes | Yes | Yes | Yes | Yes | Yes | Yes | Yes | Yes | Yes |
| Brouwers (2012) | Yes | Yes | Yes | Yes | Yes | Yes | Yes | Yes | Yes | Not clear | Yes | Yes | Yes |
| Junior (2013) | Yes | Yes | Yes | Yes | Yes | Yes | Yes | Yes | No | No | Yes | Yes | Yes |
| Rizos (2013) | Yes | Yes | Yes | Yes | Yes | Yes | Yes | Yes | Yes | Not clear | Yes | Yes | Yes |
| Romero (2013) | Yes | Yes | Yes | Yes | Yes | Yes | Yes | Yes | Yes | Not clear | Yes | Yes | Yes |
| Sun (2013) | Yes | Yes | Yes | Yes | Yes | Yes | Yes | Yes | Yes | Not clear | Yes | Yes | Yes |
| Brouwers (2014) | Yes | Yes | Yes | Yes | Yes | Yes | Yes | Yes | Yes | Not clear | Yes | Yes | Yes |
| Hotta (2014) | Yes | Yes | Yes | Yes | Yes | Yes | Yes | Yes | No | No | Yes | Not clear | Yes |
| Rodriguez-Luna (2014) | Yes | Yes | Yes | Yes | Yes | Yes | Yes | Yes | No | Not clear | No | Yes | Yes |
